# Improvement of Rat Spinal Cord Injury Following Lentiviral Vector-Transduced Neural Stem/Progenitor Cells Derived from Human Epileptic Brain Tissue Transplantation with a Self-assembling Peptide Scaffold

**DOI:** 10.1007/s12035-020-02279-5

**Published:** 2021-01-14

**Authors:** Sara Abdolahi, Hadi Aligholi, Azizollah Khodakaram-Tafti, Maryam Khaleghi Ghadiri, Walter Stummer, Ali Gorji

**Affiliations:** 1 Department of Pathobiology, School of Veterinary Medicine, Shiraz University, Shiraz, Iran; 2 Shefa Neuroscience Research Center, Khatam Alanbia Hospital, Tehran, Iran; 3 Department of Neuroscience, School of Advanced Medical Sciences and Technologies, Shiraz University of Medical Sciences, Shiraz, Iran; 4 Epilepsy Research Center, Shiraz University of Medical Sciences, Shiraz, Iran; 5 Department of Neurosurgery, Westfälische Wilhelms-Universität, Münster, Germany; 6 Epilepsy Research Center, Department of Neurology and Institute for Translational Neurology, Westfälische Wilhelms-Universität Münster, 48149 Münster, Germany; 7 Neuroscience Research Center, Mashhad University of Medical Sciences, Mashhad, Iran; 8 Department of Neuroscience, Faculty of Medicine, Mashhad University of Medical Sciences, Mashhad, Iran

**Keywords:** Trauma, Disability, Cell therapy, Scaffold, Bioengineering

## Abstract

Spinal cord injury (SCI) is a disabling neurological disorder that causes neural circuit dysfunction. Although various therapies have been applied to improve the neurological outcomes of SCI, little clinical progress has been achieved. Stem cell–based therapy aimed at restoring the lost cells and supporting micromilieu at the site of the injury has become a conceptually attractive option for tissue repair following SCI. Adult human neural stem/progenitor cells (hNS/PCs) were obtained from the epileptic human brain specimens. Induction of SCI was followed by the application of lentiviral vector-mediated green fluorescent protein–labeled hNS/PCs seeded in PuraMatrix peptide hydrogel (PM). The co-application of hNS/PCs and PM at the SCI injury site significantly enhanced cell survival and differentiation, reduced the lesion volume, and improved neurological functions compared to the control groups. Besides, the transplanted hNS/PCs seeded in PM revealed significantly higher migration abilities into the lesion site and the healthy host tissue as well as a greater differentiation into astrocytes and neurons in the vicinity of the lesion as well as in the host tissue. Our data suggest that the transplantation of hNS/PCs seeded in PM could be a promising approach to restore the damaged tissues and improve neurological functions after SCI.

## Introduction

Traumatic spinal cord injury (SCI) is a disabling neurological condition that can lead to permanent motor and sensory dysfunctions [1, 2]. Despite exhaustive investigations during the past few decades, there is still a great demand for developing novel therapeutic strategies for SCI. Stem cell replacement therapies offer great potential to restore the structure and function of damaged tissues after SCI [3, 4]. Several experimental studies have shown remarkable effects of the transplanted human-derived stem/progenitor cells, such as embryonic and mesenchymal stem cells, oligodendrocyte precursor cells, induced pluripotent stem cells, fetal Schwann cells, neural stem cells, dental pulp stem cells, and adipose-derived stem cells, on the functional recovery after SCI, confirming that under certain conditions, these cells can proliferate and differentiate into various neuroglial lineages [5–8]. The human neural stem/progenitor cells (hNS/PCs) display multipotency with high proliferation and differentiation capacities, secrete multiple trophic factors, and exhibit neuroprotective and neuroregenerative properties [9]. Thus, transplantation of hNS/PCs into the injury site could be a conceptually optimal stem cell-based therapy for SCI [10]. However, using hNS/PCs for stem cell therapy may be limited by the low survival rate of grafted cells due to the mechanical damage during cell transplantation, destructive inflammatory response, and/or absence of appropriate microenvironment [11–13]. Therefore, creating a suitable microenvironment is crucial for better survival, proliferation, and migration of hNS/PCs. Scaffold-based tissue engineering is an appropriate platform to support cellular proliferation, differentiation, attachment, migration, and survival [14]. PuraMatrix (PM) is a 16-amino acid synthetic peptide hydrogel (RADA16) that exhibits high water solubility, controllable biodegradation, biocompatibility, flexibility, and hydrophilicity with low cellular toxicity [15, 16]. PM supported attachment, proliferation, differentiation, and survival of hNS/PCs and improved the functional outcomes after brain injury in an experimental animal model [17].

As the host micromilieu plays a crucial role in stem cell fate determination, the differentiation, maturation, and migration ability of hNS/PCs after transplantation is a decisive factor in determining their integration with host tissues [8]. Lentiviral gene transfer could be used as a promising tool to study the differentiation of hNS/PCs into various cell fates as well as to evaluate the cell migration into healthy host tissue in various pathological conditions, including SCI [18–20]

Epilepsy surgery offers a unique opportunity to achieve hNS/PCs from the resected brain tissues of patients suffering from medically refractory epilepsy [21]. The hNS/PCs derived from epileptic brain tissues have the essential characteristics of neuroglial cells, which may provide an ideal source of autologous stem cell for replacement and restorative therapies [17, 22, 23]. In this study, we have investigated the proliferation and differentiation capacities as well as functional efficacy of lentiviral vector-mediated green fluorescent protein (GFP)-labeled hNS/PCs seeded in PM in a rat model of SCI. The behaviors of transduced hNS/PCs in the vicinity of the lesion as well as in the healthy host tissue were evaluated.

## Materials and Methods

All experiments were performed in accordance with the National Institute of Health Guide for the Care and Use and were approved by the Ethics Committee of Shefa Neuroscience Research Center, Tehran, Iran. After receiving informed consent, fresh tissue was obtained from the temporal lobe of two patients with mesial temporal lobe epilepsy during surgical intervention. The past medical history of patients is shown in Table 1.

**Table 1:** The past medical history of patients

Case	Gender	Age (year)	Age of onset	Duration of epilepsy (year)	Seizure frequency	AED/drug history	Pathology/imaging	Seizure type
1	Male	28	14	14	1–2/weekly	Valproic acid, Carbamazepine, Gabapentin	Hippocampal sclerosis	GS*
2	Male	28	10	18	2–3/weekly	Carbamazepine, Phenytoin	Hippocampal sclerosis	GS

*GS generalized seizures

### Experimental Design

**Fig. 1:**
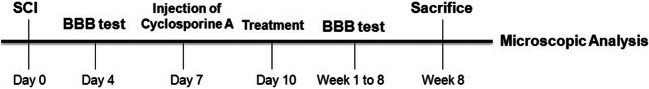
An overview of experimental design. The induction of spinal cord injury (SCI) was followed by the transplantation of green fluorescent protein–labeled adult human neural stem/progenitor cells (hNS/PCs) obtained from the epileptic human brain with PuraMatrix scaffold after 10 days. The Basso, Beattie, and Bresnahan (BBB) locomotor score was accessed after the induction of SCI. Eight weeks after treatment, rats were sacrificed and histopathological and immunohistochemical studies were performed

A contusion model of SCI was performed in rats. The subcutaneous application of cyclosporine A (1 mg/100 g body weight/24 h) was begun on day 3 before the cell transplantation and continued for another 2 days [24]. All animals then received phosphate-buffered saline (PBS), PM, hNS/PCs, or PM + hNS/PCs on day 10 after the induction of SCI. Assessments of recovery of motor function were tested on day 4 after the induction of the SCI and 8 times (weekly) during the post-treatment period using the Basso, Beattie, and Bresnahan (BBB) locomotor rating scale. Animals were sacrificed for microscopic analysis at week 8 (Fig. 1). All animal care programs and surgical procedures were carried out with strict accordance and dedicated facilities.

Thirty-two adult male Wistar rats (220–250 g) were maintained in the animal house of the Shefa Neuroscience Research Center. The animals were housed in controlled condition (12-h light/dark cycle 21 ± 2 °C; 40–60% air humidity) with unrestricted access to standard laboratory food and water, and then divided randomly into four treatment groups (8 rats per group): (i) Control group: SCI rats with PBS treatment, (ii) PM group: SCI rats with 0.15% PM treatment, (iii) hNS/PCs group: SCI rats with 1 × 10^4^ green fluorescent protein (GFP)-labeled hNS/PCs treatment, and (iv) PM + hNS/PCs group: SCI rats with 1 × 10^4^ GFP-labeled hNS/PCs + PM. All data assessments were conducted blinded to the group allocation.

### Preparation of hNS/PCs

The resected epileptic brain tissues were washed twice in cold PBS containing 1% penicillin/streptomycin. After dissection of tissues, enzymatic digestion and mechanical dissociation were performed. Briefly, tissue sections were treated with Accutase (Gibco, Germany) for 15 min at room temperature and the suspension was repeatedly pipetting up and down. The enzyme was replaced with an equal volume of the fresh medium to stop the enzymatic reactions. Then, the suspension was centrifuged for 5 min at 110 g and the supernatant discarded and finally dissolved in 1–2 ml of Dulbecco’s modified Eagle’s medium/F12 (DMEM/F12; Gibco, Germany). The cultures were maintained as neurospheres in uncoated tissue culture flasks in a serum-free medium. For cell culture and expansion, the medium contained DMEM /F12 supplemented with 3% B27 supplement (Invitrogen, USA), 0.5% N2 supplement (Invitrogen, USA), 10 ng/ml basic fibroblast growth factor (bFGF; Millipore, Germany), 20 ng/ml epidermal growth factor (EGF; Miltenybiotech, Germany), 2 μg/ml heparin (Sigma, Germany), 1% penicillin/streptomycin, and 1% glutamine (Invitrogen, USA). The culture medium was replaced with a fresh medium every 3 days. The neurospheres were expanded by passaging into fresh medium every 7 days. The expanded neurospheres were visualized by an inverted microscope. The numbers of the neurospheres were analyzed in six randomly selected well from 96-well plates using the ImageJ software.

### Immunofluorescence Staining

Immunofluorescence assay was done to identify the expression of markers in the isolated cells. After discarding the cell growth medium, fixation was carried out with 4% paraformaldehyde in PBS for 20 min at room temperature. Permeabilization of cells was conducted using 0.2% Triton X-100, and 5% normal goat serum (NGS; Abcam). Then, the cells were exposed to the primary antibody against the glial fibrillary acidic protein (GFAP) (1:200 diluted in PBS; Millipore), nestin (1:50 diluted in PBS; Santa Cruz), and Sox2 (1:100 diluted in PBS; Santa Cruz) as markers of adult neural stem/progenitor cells. Subsequently, the cells were incubated with secondary antibodies, including goat anti-rabbit IgG (FITC, 1:1000 diluted in PBS; Abcam), goat anti-mouse (FITC, 1:1000 diluted in PBS; Abcam), and rabbit anti-goat (FITC, 1:1000 diluted in PBS; Sigma) for 2 h at room temperature in the dark. Cell nuclei were counterstained with 4′,6-diamidino-2-phenylindole (DAPI). Cells were photographed using fluorescent microscopy (Olympus, Japan). Samples were incubated with secondary antibodies alone as negative control.

### hNS/PCs Transduction by Lentiviral Particle

Transduction of hNS/PCs with recombinant lentiviral particles was performed to track them after transplantation. The lentiviral transduction process has been described previously [20]. Briefly, recombinant viruses were produced using three lentiviral plasmids: pCDH, psPAX2, and pMD2.G. The pCDH and psPAX2 plasmids contain GFP and gag and pol genes, respectively. The pMD2.G plasmid was used as an envelope plasmid encoding the VSV G surface protein. The human embryonic kidney 293T cell (HEK-293T) line was applied as a host for virus packaging (National Cell Bank, Pasteur Institute, Iran). The plasmids were simultaneously transfected into 80–90% confluent HEK-293T cells by Lipofectamine 3000 reagent (Invitrogen, USA). Then, the lentiviral particles were used to transduce hNS/PCs. The hNS/PCs were seeded in a T25 culture flask until they reached 80% confluency. The expression of GFP was assessed after 3 days. The multiplicity of infection (MOI) was used as a parameter for the prediction of cell transduction [20].

### Spinal Cord Contusion Model

All animals were anesthetized intraperitoneally (i.p.) by ketamine (80 mg/kg) and xylazine (10 mg/kg) during the surgical process and their body temperature was maintained at 37 °C. SCI was performed according to the weight compression method. The lack of feedback to noxious stimulation was considered a symptom of general anesthesia. After that, the back region of the rat was shaved, and the skin was disinfected. After the exposure of the spine by incision, laminectomy was done at the T9 and the T10 segments. Spinal cord compression was applied using 35 g weight placed on the dura for 15 min. The weight with concave shape and area of 6.6 mm^2^ (3.0–2.2 mm) was used to provide equal pressure distribution on the spinal cord.

After the injury, the device was removed and the muscle, fascia, and skin were sutured separately, and then i.p. injection of lactated Ringer’s solution was used to treat dehydration. Gentamicin (6 mg/kg) was given by intramuscular injection for 3 days after surgery. Manual bladder expression was done to remove urine twice daily until recovery.

### Treatments

Ten days following induction of SCI, animals were anesthetized as described previously and the lesion site was re-exposed. 100 μl of PBS, 100 μl of PM (0.15%), 100 μl of PBS containing 1 × 10^4^ GFP-labeled hNS/PCs, or 100 μl of PBS containing 1 × 10^4^ GFP-labeled hNS/PCs + PM was prepared. The solutions (in 3 equal volumes) were injected in the center, rostral, and caudal parts of the injury site with a distance of about 3 mm within 5 min using a 26-g Hamilton syringe. The tip of the needle was inserted to a depth of 1 mm for the injection of solutions. After transplantation, all animals survived for 8 weeks.

### Behavioral Assessments

The recovery of the locomotor function was assessed by using the BBB motor performance test.

The BBB score was assessed 4 days after the induction of SCI. To standardize the extent and severity of the SCI, rats with hind limb score differences greater than 4 were removed from the study. The treatment was performed on day 10 after the induction of SCI. BBB assessments were done for 8 consecutive weeks after the treatment. BBB scores were measured to categorize the combinations of left and right hind limb movements, trunk stability, stepping and paw position, consistent toe clearance, and tail position. Briefly, each rat was observed and recorded for 4 min in an open field under blind conditions. Left and right hind limb movements were individually scored. BBB scores were evaluated the average of both hind limbs in individual animals.

### Tissue Preparation

Eight weeks after the treatment, animals were anesthetized (as described previously) and transcardially perfused with 1X PBS containing heparin (pH 7) followed by cold 4% paraformaldehyde. The segments of the spinal cord containing the graft were rapidly dissected out and immediately fixed in 4% paraformaldehyde for 24 h. After tissue processing, tissues were embedded in paraffin and cut at 5-μm thickness.

### Assessment of Lesion Volume

To assess the lesion volume formed after SCI, a series of sections repeated at 100-μm intervals were dyed with hematoxylin and eosin (H&E) staining. Finally, the images were taken from each sample and then analyzed using the Axio-Vision 4 software. The total lesion volume was calculated by the following formula: 0.5*D*(*A*1 + *A*_*n*_) + *D* (*A*2 + *A*3 +…+ *A*_*n*−1_), where *A* is the area of the cavity and *D* is the distance between sections [25].

### Immunohistochemistry Assay

Before immunofluorescent staining, the deparaffinization process was performed to remove the penetrated paraffin from the tissue. The slides were immersed in a sodium citrate buffer at pH 6.0 and heat samples near boiling in a water bath for 10 min. Sections were then permeabilized by treating with 0.25% Triton X-100 (30 min, room temperature), and blocked for 1h by 0.5% BSA (Sigma, Germany) with 5% NGS. Sections were then exposed overnight with the diluted primary antibodies, including mouse anti-nestin (1:50; Abcam, UK) for neural stem/progenitor cells, mouse anti-GFAP (1:100; Sigma, Germany) for astrocytes, and rabbit anti-doublecortin (1:100; Santa Cruz, Germany) for immature neurons at 4 °C and rinsed in TBS three-time before the application of secondary antibody. Alexa Fluor 647 goat anti-mouse (1:600; Abcam, UK) and Alexa Fluor 647-goat anti-rabbit (1:500; Abcam, UK) were applied for 60–90 min at room temperature. In negative controls, the primary antibodies were omitted. The slides were then washed with TBS and nuclei were stained with DAPI (Sigma-Aldrich, Germany). The sections mounted with glycerol buffer under coverslips. The percentages of nestin, GFAP, and doublecortin positive cells (GFP-positive cells) per image field were calculated relative to the total number of GFP-positive cells in hNS/PCs and hNS/PCs + PM groups.

### Statistical Analysis

Statistical analyses of the present study were conducted with SPSS version 22. All data are expressed as the mean ± SEM. One-way analysis of variance (ANOVA) test was used to analyze differences among all groups, and post hoc tests were performed with Tukey’s test. An independent *t*-test was carried out to compare one variable between two groups. The *P* values of less than 0.05 were considered significant.

## Results

### Isolation, Characterization, and Transduction of hNS/PCs

**Fig. 2:**
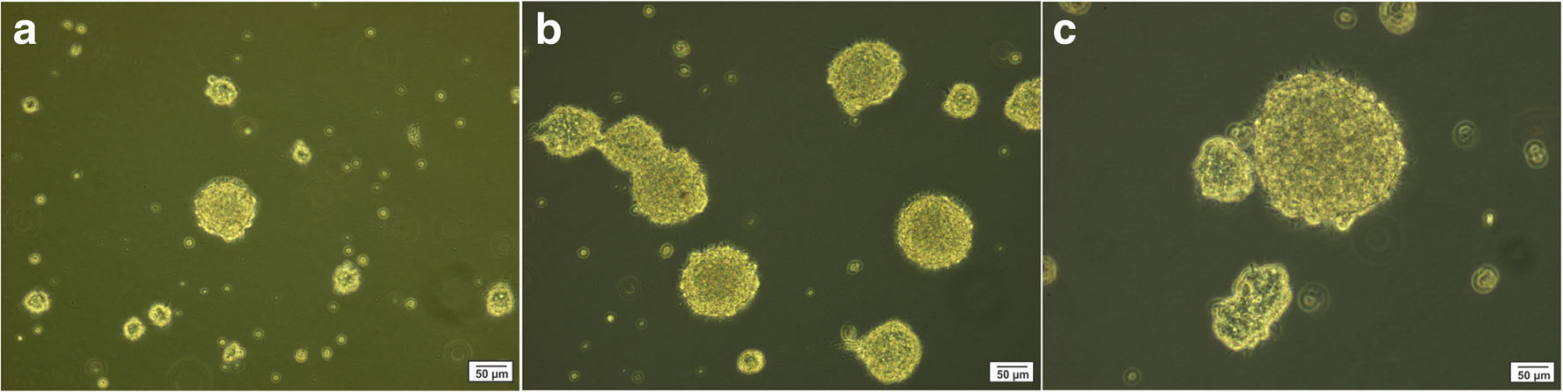
Representative phase-contrast images of neurosphere clusters derived from human neural stem/progenitor cells after 4 days (**a**), neurospheres with the translucent center after 7 days (**b**), and larger neurospheres with the dark center after 14 days (**c**) in culture are shown

**Fig. 3:**
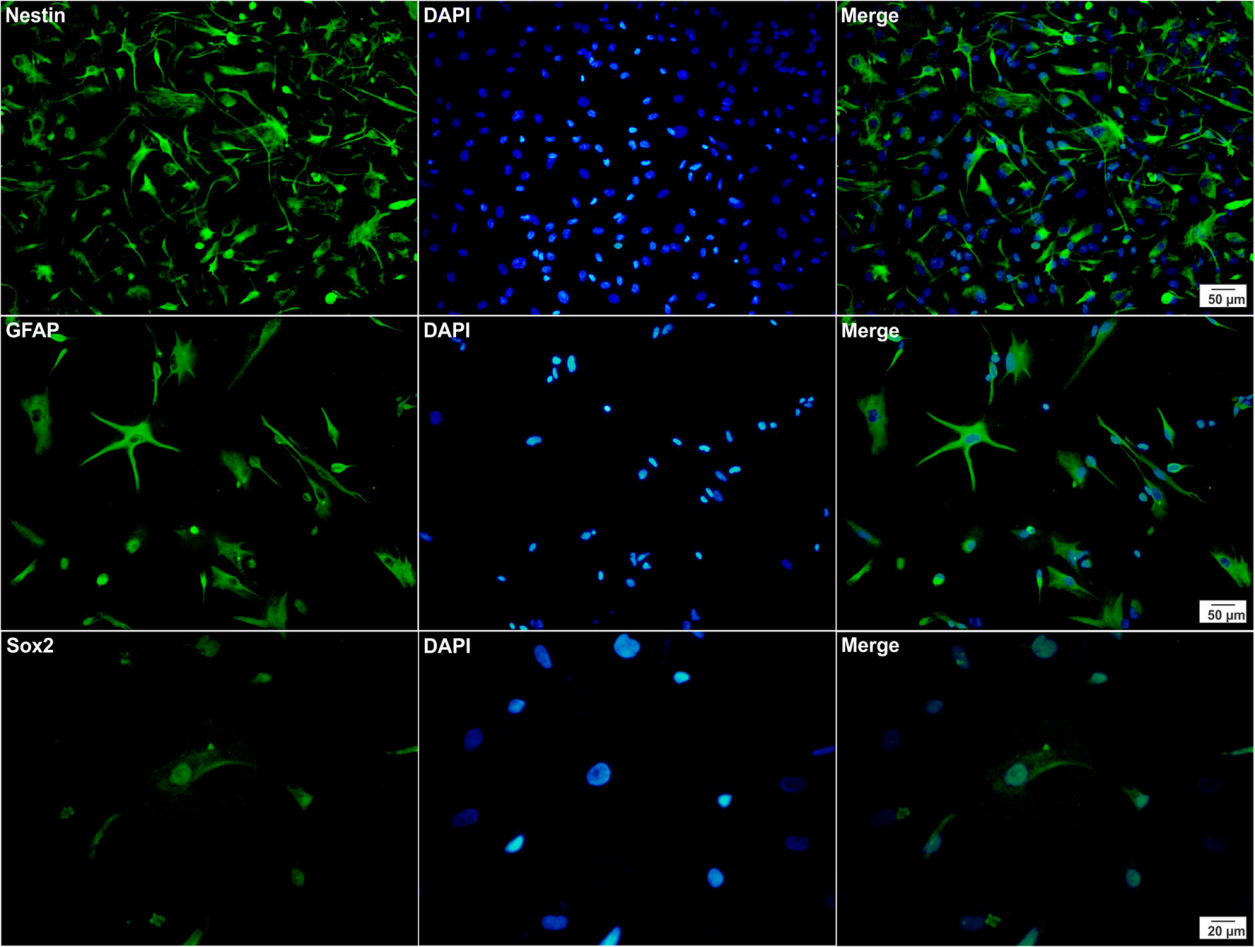
Immunocytochemical studies of human neural stem/progenitor cells were performed to analyze the expression of nestin, GFAP, and Sox2 markers. All markers are shown in green (left column) and the cell nuclei (counterstained with DAPI) are shown in blue (middle column). The merged images are shown in the right column

**Fig. 4:**
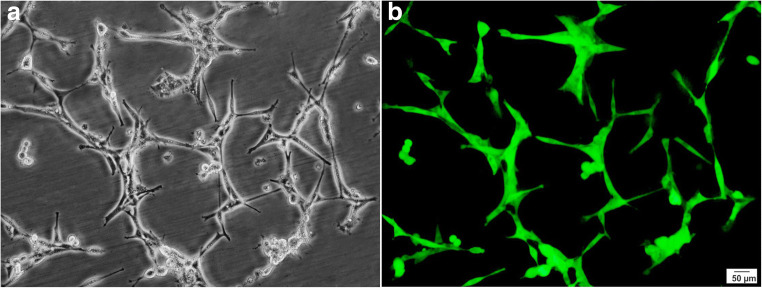
Representative photomicrographs of human neural stem/progenitor cells after transduction by lentiviruses containing green fluorescent protein taken through a light microscope (**a**) and a fluorescent microscope (**b**)

Resected brain tissues from two patients during epilepsy surgery were dissociated into individual cells and grown under conditions that promote the neurosphere-forming. On the 7th day of cell culture, the floating neurospheres were harvested, and subsequently, the expansion process was carried out to the 2^nd^ and 3^rd^ passage (Fig. 2). Immunofluorescence analysis after the 3^rd^ passage has shown that the neurosphere-derived single cells strongly expressed nestin, GFAP, and Sox2, the markers of neural stem/progenitor cells (Fig. 3). Furthermore, using transient co-transfection of the HEK-293T cells with three plasmids (pCDH, psPAX2, and pMD2.G), vectors were generated at titers of ∼ 50 × 10^6^ transducing units/ml. hNS/PCs were cultured in T25 flasks until the cells reached a confluence of 80% and then were transduced with GFP-expressing lentiviruses with MOI of ∼ 51 (Fig. 4) [20].

### Functional Recovery After SCI

To assess the functional recovery of rat hind limbs, the behavioral test was performed on day 4 after the induction of SCI and then weekly up to 8 weeks after the treatment. There was no significant difference was observed in the BBB scores 4 days after the induction of SCI.

**Fig. 5:**
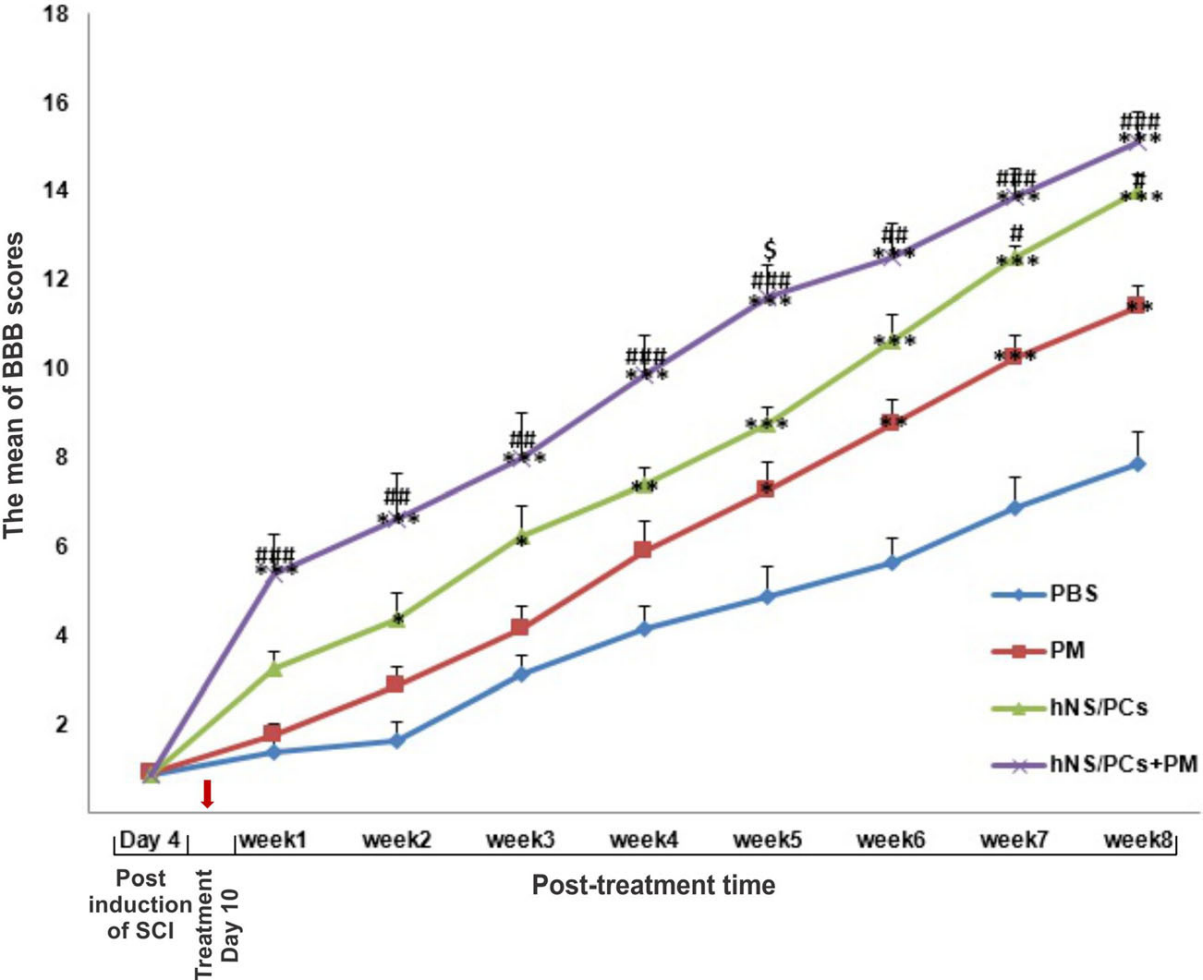
Alterations of the Basso, Beattie, and Bresnahan (BBB) locomotor rating scale following spinal cord injury (SCI) induction. BBB scores in various groups are shown 4 days after the induction of SCI as well as eight weeks after treatment. There was no significant difference in BBB scores between all groups at day 4 after the induction of SCI. Changes of BBB score after administration of phosphate-buffered saline (PBS), PuraMatrix (PM), and transplantation of human neural stem/progenitor cells (hNS/PCs) with and without PM during 8 weeks after treatment. Data are represented as the mean ± SD. * indicates *P* < 0.05 vs. the PBS group, *** indicates *P* < 0.001 vs. the PBS group, # indicates *P* < 0.05 vs. the PM group, ### indicates *P* < 0.001 vs. the PM group, and Ś indicates *P* < 0.05 vs. the hNS/PCs group

The BBB scores of rats exhibited a gradual upward trend at all time points after the treatment in different groups. However, treatment with hNS/PCs or hNS/PCs + PM resulted in a greater and faster functional recovery compared to the control groups. The motor function recovery of the hNS/PCs + PM, hNS/PCs, and PM groups significantly improved 1, 2, and 5 weeks after the treatment, respectively, compared to the PBS group (*P* < 0.05). The application of hNS/PCs led to a significant improvement of functional recovery after 7 weeks of the treatment compared to the PM group (*P* < 0.05). Moreover, treatment with hNS/PCs + PM has demonstrated a significantly higher BBB score after 1 week of the treatment compared to the PM group (*P* < 0.05). The application of hNS/PCs + PM has shown a significantly higher BBB score on week 5 after the treatment compared to the hNS/PCs group (*P* < 0.05; Fig. 5).

### Measurement of the Lesion Volume

**Fig. 6:**
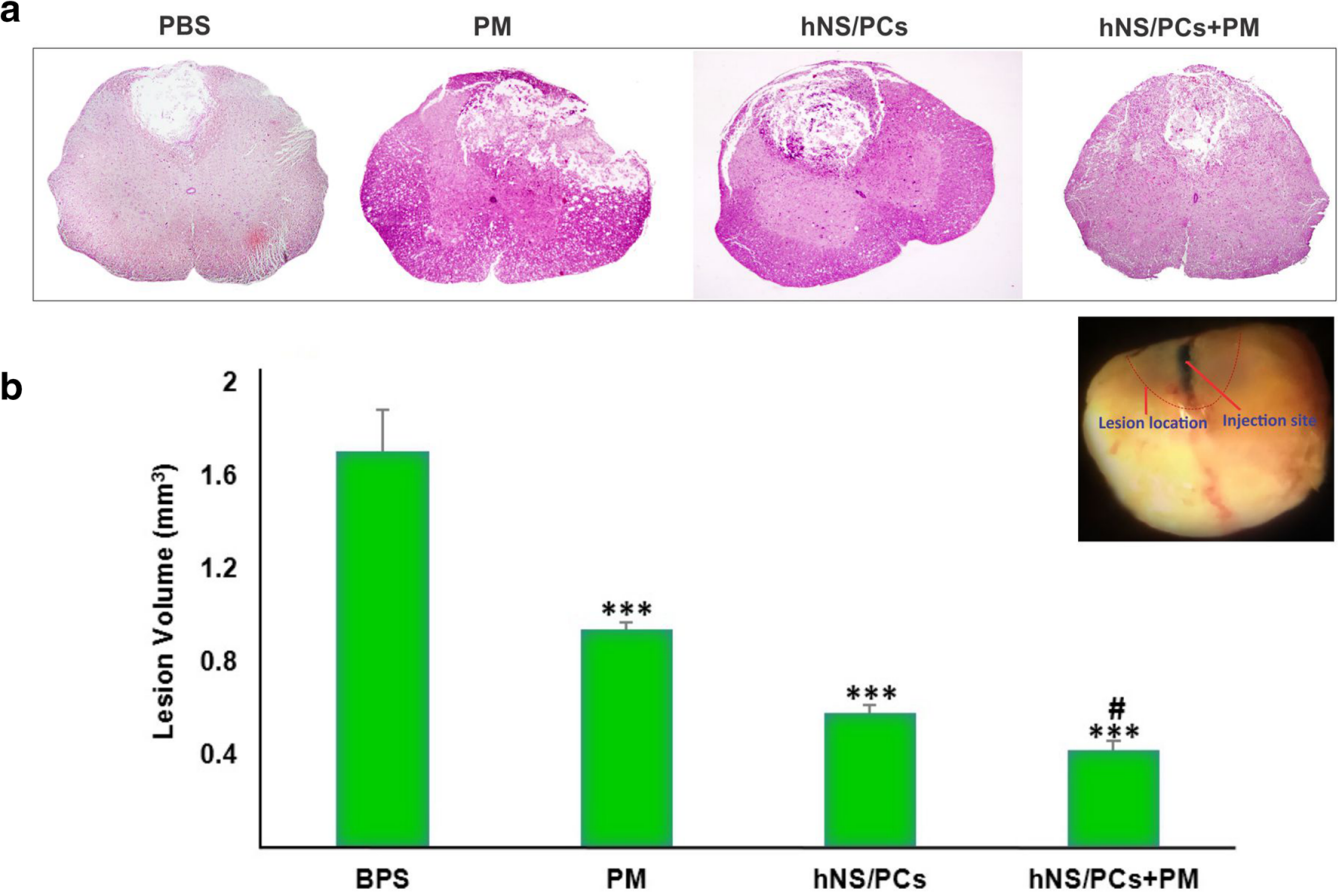
Assessment of the lesion volume 8 weeks after treatment in different groups using H&E staining. Representative photomicrographs of coronal sections of the spinal lesion in different animal groups treated with phosphate-buffered saline (PBS), PuraMatrix (PM), and human neural stem/progenitor cells (hNS/PCs) with and without PM (**a**). Quantitative analysis of the lesion volume in different experimental groups (**b**). We observed a significant decrease in the lesion volume of the PM group, hNS/PCs group, and hNS/PCs+PM group compared with the PBS groups. Note that the lesion volume was significantly lower in the hNS/PC + PM group compared to the PM group. Data are represented as the mean ± SD. *** indicates *P* < 0.05 vs. the PBS group and # indicates *P* < 0.05 vs. the PM group

The induction of SCI led to the formation of a cavity at the injury site. The lesion volume was measured after H&E staining. The mean lesion volume was significantly decreased in the PM (0.94 ± 0.06 mm^3^), hNS/PCs (0.58 ± 0.04 mm^3^), and hNS/PCs + PM (0.42 ± 0.01 mm^3^) groups compared to the PBS group (1.71 ± 0.16 mm^3^; *P* < 0.05). Besides, we observed a significant decrease in the lesion volume in the hNS/PCs + PM group compared to the PM groups (*P* < 0.05; Fig. 6). These data indicate a significant reduction in SCI-induced cavity formation after transplantation of hNS/PCs + PM compared with the PM group.

### Survival of the Transplanted Cells

**Fig. 7:**
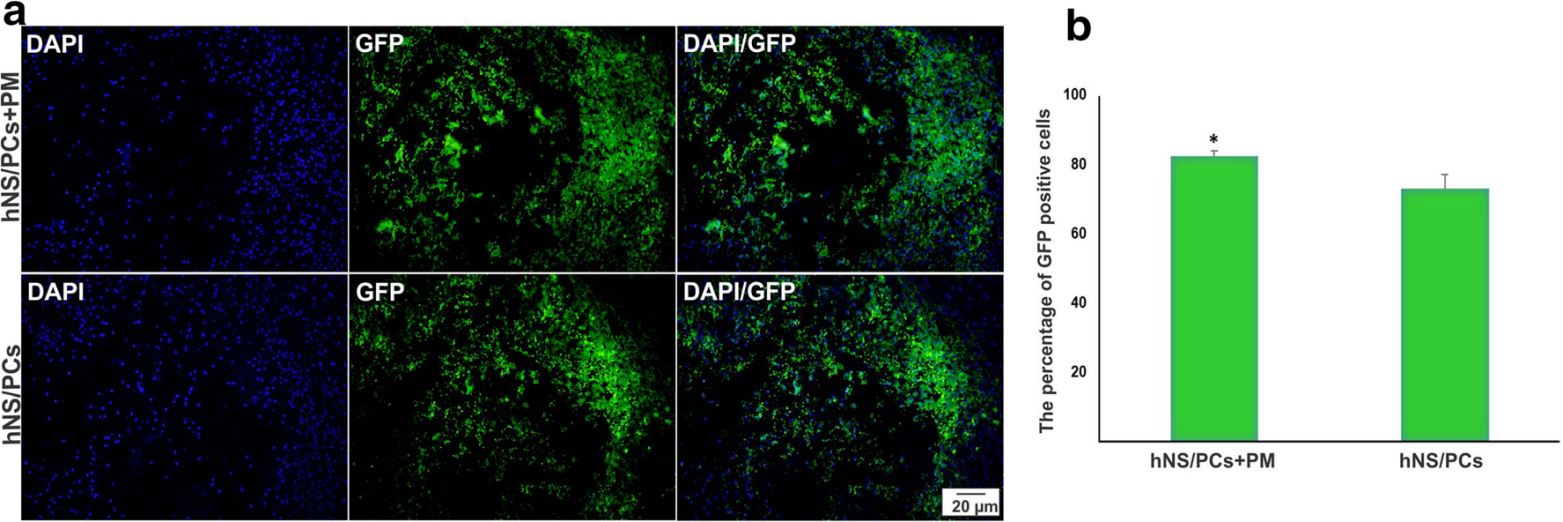
The expression of green fluorescent protein (GFP)-labeled transplanted human neural stem/progenitor cells (hNS/PCs) with and without PuraMatrix (PM) in the spinal cord lesion site. Representative immunofluorescence images of the expression of DAPI (shown in blue, left column), GFP-positive hNS/PCs (shown in green, middle column), and the merged view (right column) of the hNS/PCs and hNS/PCs + PM groups (**a**). The mean percentage of GFP-positive cells 8 weeks after transplantation of hNS/PCs + PM and hNS/PCs in the lesion site of spinal cord injury (**b**). Data are represented as the mean ± SD. * indicates *P* < 0.05

The long-term survival of hNS/PCs was significantly increased when seeded in the PM. Abundant GFP-positive transplanted cells were observed in the vicinity of the injury in the hNS/PCs + PM and hNS/PCs groups. The percentage of GFP-positive transplanted cells was significantly higher in the hNS/PCs + PM group (82.4 ± 1.6%) compared to the hNS/PCs group (72.9 ± 4.1%, *P* < 0.05; Fig. 7).

### The Fate of hNS/PCs After Cell Transplantation

**Fig. 8:**
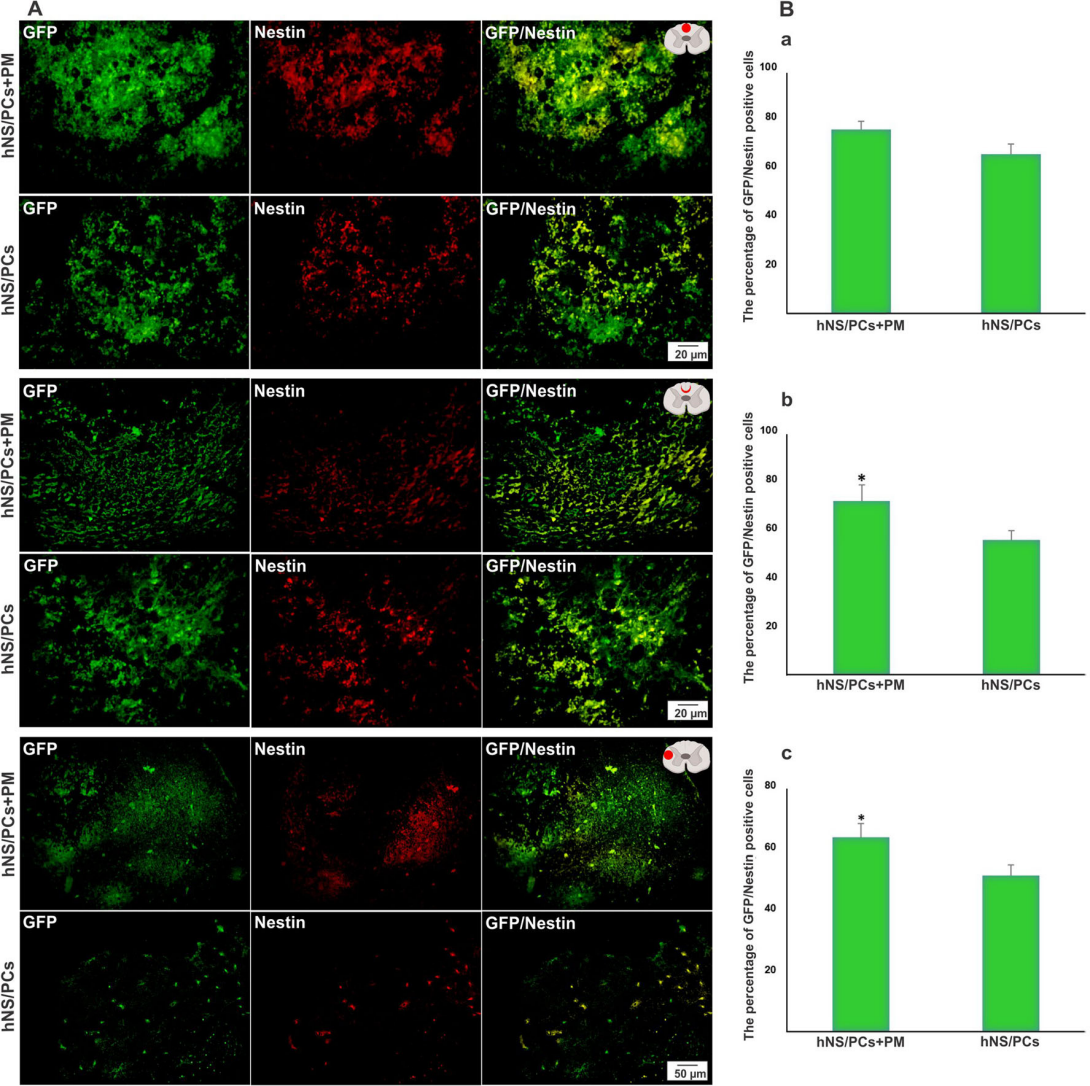
The expression of nestin (a neural stem/progenitor cell marker) within the lesion site, in the perilesional region, and in the healthy host tissue (extralesional region) 8 weeks after transplantation of human neural stem/progenitor cells (hNS/PCs) with and without PuraMatrix (PM) in rats with spinal cord injury. Representative immunofluorescence images of the expression of green fluorescent protein (GFP; shown in green, left column), nestin (shown in red, middle column), and the merged images (right column) of the hNS/PCs and hNS/PCs + PM groups in three investigated areas (**a**). The mean percentage of GFP-nestin-positive cells in the hNS/PCs + PM and hNS/PCs groups in three studied regions (**b**). Data are represented as the mean ± SD. * indicates *P* < 0.05

**Fig. 9:**
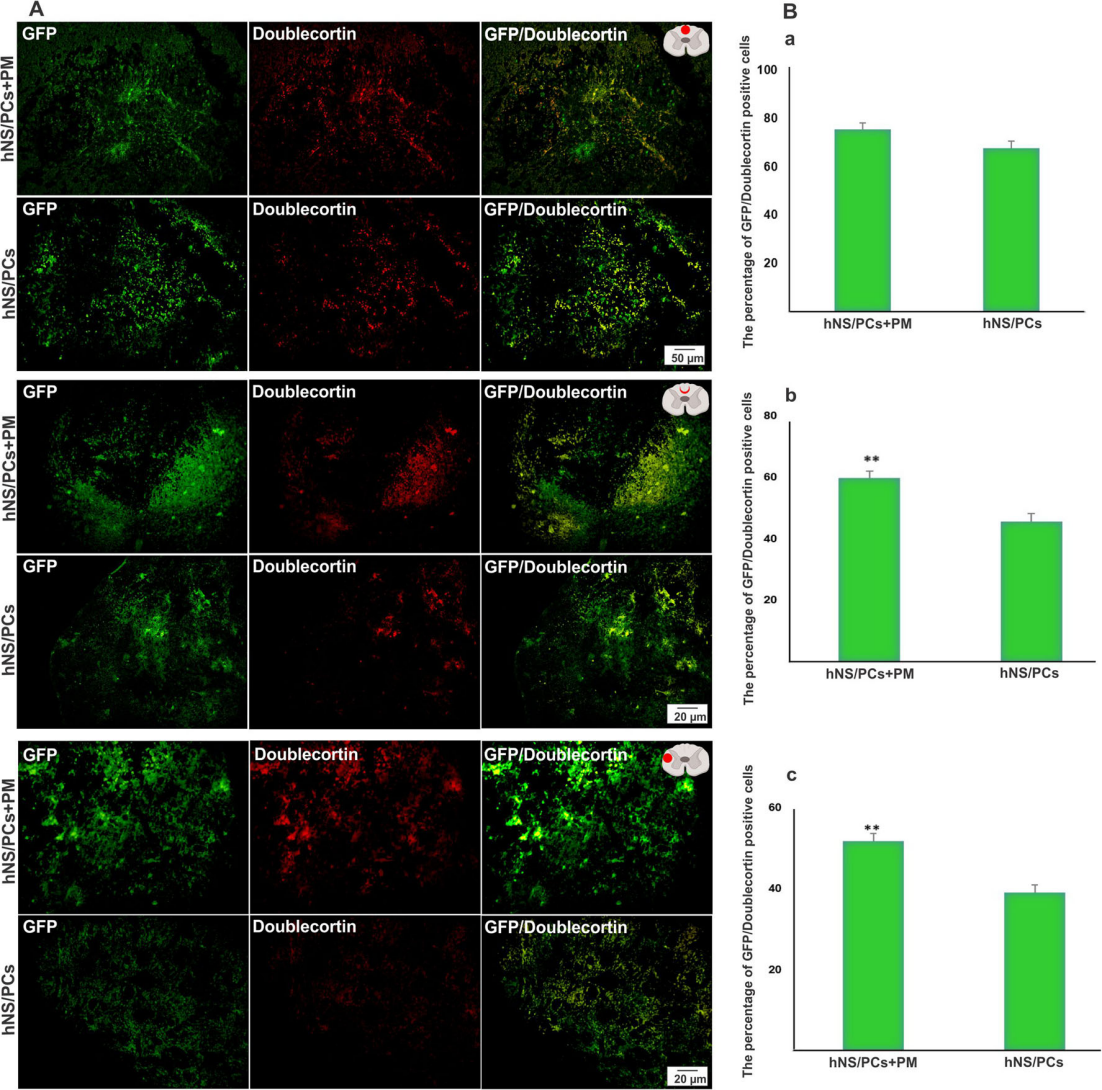
The expression of doublecortin (a marker of neuronal migration) within the lesion site, in the perilesional region, and in the healthy host tissue (extralesional region) 8 weeks after transplantation of human neural stem/progenitor cells (hNS/PCs) with and without PuraMatrix (PM) in rats with spinal cord injury. Representative immunofluorescence images of the expression of green fluorescent protein (GFP; shown in green, left column), doublecortin (shown in red, middle column), and the merged images (right column) of the hNS/PCs and hNS/PCs + PM groups in three investigated areas (**a**). The mean percentage of GFP-doublecortin positive cells in the hNS/PCs + PM and hNS/PCs groups in three studied regions (**b**). Data are represented as the mean ± SD. * indicates *P* < 0.05

**Fig. 10:**
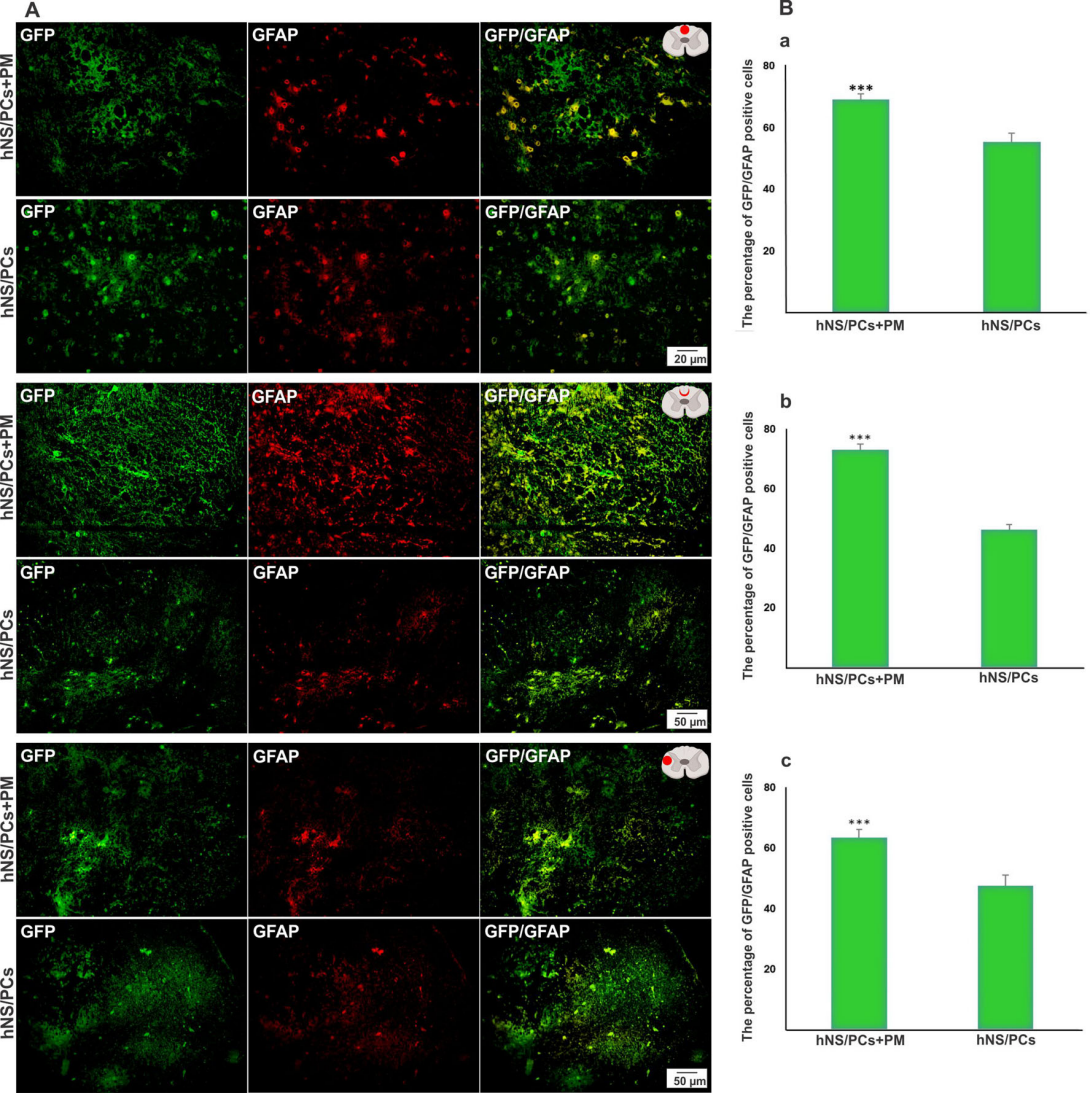
The expression of GFAP (an astrocyte marker) within the lesion site, in the perilesional region, and in the healthy host tissue (extralesional region) 8 weeks after transplantation of human neural stem/progenitor cells (hNS/PCs) with and without PuraMatrix (PM) in rats with spinal cord injury. Representative immunofluorescence images of the expression of green fluorescent protein (GFP; shown in green, left column), GFAP (shown in red, middle column), and the merged images (right column) of the hNS/PCs and hNS/PCs + PM groups in three investigated areas (**a**). The mean percentage of GFP-GFAP positive cells in the hNS/PCs + PM and hNS/PCs groups in three studied regions (**b**). Data are represented as the mean ± SD. * indicates *P* < 0.05

To explore the migratory ability of transplanted cells and their capacity to differentiate into neuroglial cells in vivo, the expressions of nestin, GFAP, and doublecortin in intralesional, perilesional, and extralesional sites were assessed in the transplanted cells. The mean percentage of GFP-nestin, GFP-doublecortin, and GFP-GFAP positive cells in the perilesional and extralesional regions was significantly higher in the hNS/PCs + PM group compared to the hNS/PCs group (*P* < 0.05; Figs. 8, 9, and 10). Furthermore, the expression of GFP-GFAP positive cells in the intralesional region was significantly higher in the hNS/PCs + PM group compared to the hNS/PCs group (*P* < 0.05; Fig. 10). There were no significant differences between the mean percentage of GFP-nestin and GFP-doublecortin expressions in the intralesional region between the hNS/PCs + PM and hNS/PCs groups (Figs. 8 and 9). These data indicate the facilitating role of the PM in the migration and differentiation of hNS/PCs within the lesion site as well as the host tissue.

## Discussion

The long-term GFP expression in hNS/PCs using lentiviral vectors allowed us to track the cells within the injured spinal cord and healthy host tissues as well as to monitor hNS/PCs behavior following transplantation. Our data indicate the efficacy of hNS/PCs derived from human epileptic mesial temporal lobe tissues combined with PM in facilitating SCI repair and improvement of neurological functions. Furthermore, the data demonstrated the multilineage differentiation capacity of hNS/PCs and the ability to migrate within the lesion site as well as in the healthy host tissues 8 weeks after transplantation. The peptide hydrogel PM significantly promotes the differentiation and migration of hNS/PCs.

The strong expressions of nestin, GFAP, and Sox2 in neurosphere-derived single cells confirmed the potential of harvesting neural stem/progenitor cells from the adult human brain. These cells proliferated as neurospheres in vitro and differentiated into neural and glial lineage in vivo. In keeping with our results, several investigations have shown that adult neural stem cells can proliferate and differentiate into various cell lineages, including astrocytes and neurons [26–28]. In addition to the reduction of cortical lesion volume and improvement of neurological function, our previous study revealed that the administration of rat NS/PCs seeded in PM led to increased cell survival and production of three main neural cell types, i.e., neurons, oligodendrocyte, and astrocytes [22]. The presence of astrocytes in the white matter of the injury site in the early phase of SCI is essential for tissue regeneration [29]. Significant and time-dependent enhancement of star-like astrocytes as well as a centripetal migration to the lesion epicenter after SCI was associated with potent reparative and regenerative effects [30, 31], possibly through their ability to respond to inflammatory stimulants and phagocytic capacity [32]. Our study indicates that using hNS/PCs with PM significantly increased the presence of astrocyte-like cells in the lesion site and host tissue, which was accompanied by a greater functional recovery. Previous studies have indicated that the PM is a potential candidate for nervous tissue engineering due to its biocompatible, conductive, and non-biodegradable nature [33, 34]. Application of PM alone into the SCI lesion site resulted in an increased number of regenerating axons, a significant reduction of lesion volume, and improvement of functional recovery [35]. PM potently supports neural differentiation and long-term survival and maturation of stem cells [33, 36]. Moreover, PM provides a suitable 3D microenvironment for NS/PCs to differentiate into neurons and astrocytes and promotes nerve regeneration and myelination as well as axon regrowth across the SCI lesion site [37]. In addition, the nanofiber density and average pore size of PM provides a suitable 3D microenvironment for hNS/PCs migration toward host tissue [15, 23]. Besides, PM stimulates the differentiation of immature cells into mature neurons and astrocytes through the modulation of the genes implicated in neural cell differentiation and maturation [38]. Furthermore, PM provides a permissive 3D environment for regulating neural stem cell migration and recruitment to the sites of injured tissue and host tissues [24, 39]. In addition to our results, previous investigations have shown that PM can remarkably enhance the number of surviving transplanted cells [40, 41]. Increased survival and migration of engrafted hNS/PCs in PM could be due to significantly improved biocompatibility and integration with the host tissue, which promotes the regeneration of damaged spinal cord tissue [24, 39]. Hydrogel scaffolds play a shielding role against the host immune system and exhibit anti-gliosis and anti-inflammatory properties when fused with host tissue [42–44].

Adult neural stem cells are promising alternatives to current cell replacement therapies for neurodegenerative disorders [45, 46]. hNS/PCs obtained from epileptic brain tissues have been suggested as a favorable source of adult neural stem cells which can be used for the treatment of various neurological disorders [28]. Several studies have pointed to the proliferative and multipotent properties of hNS/PCs obtained from resected brain tissues of patients with medically refractory epilepsy [17, 26–28]. These hNS/PCs have the potential to differentiate into both neuronal and glial cells associated with both excitatory and inhibitory synaptic plasticity [47]. Functional synaptic connections between the host and transplant neural cells are essential for the restoration of the spinal cord circuits, improvement of nerve conduction, and promotion of locomotor function recovery [48]. Our study has revealed that lentivirus vectors can mediate the efficient and stable transduction of these stem cells in vivo. This finding enables us to monitor the proliferation and fate of hNS/PCs as well as their integration into the host tissue, which has considerable promise to ascertain the mechanisms of hNS/PCs-mediated seizure suppression in drug-resistant epilepsy [49, 50].

## Conclusion

Surgical treatment of intractable epilepsy provides a promising adult human neural stem cell source, which its transplantation with a 3D peptide hydrogel promoted functional recovery, reduced lesion volume, and enhanced cell migration and differentiation ability at the SCI lesion site in rats. Further investigations are warranted to understand the mechanisms involved in the observed functional outcome improvement; particularly, the main factors mediated the integration of hNS/PCs into existing neural circuits in the spinal cord.

## Data Availability

Data are available from the authors upon request.
